# Effects of *Malassezia globosa* on the Expression of Thymic Stromal Lymphopoietin and Differentiation of T Helper Cells in MC903-Induced Atopic Dermatitis Mouse Model

**DOI:** 10.1155/ijm/3586621

**Published:** 2025-04-22

**Authors:** Xin Zhou, Zhuanggui Cheng, Qintai Yang, Han Ma, Yang Xie, Zhe Xu, Jun Xia, Jian Chen, Chun Lu, Peiying Feng

**Affiliations:** ^1^Department of Dermatology, The Third Affiliated Hospital of Sun Yat-sen University, Guangzhou, China; ^2^Department of Allergy, The Third Affiliated Hospital of Sun Yat-sen University, Guangzhou, China; ^3^Department of Paediatrics, The Third Affiliated Hospital of Sun Yat-sen University, Guangzhou, China; ^4^Department of Otolaryngology, The Third Affiliated Hospital of Sun Yat-sen University, Guangzhou, China; ^5^Department of Dermatology, The Fifth Affiliated Hospital of Sun Yat-sen University, Zhuhai, China; ^6^Department of Dermatology, Beijing Children's Hospital, Capital Medical University, National Center for Children's Health, Beijing, China; ^7^Department of Dermatology, Shunyi Maternal and Children's Hospital of Beijing Children's Hospital, Beijing, China; ^8^Department of Dermatology, The Seventh Affiliated Hospital of Sun Yat-sen University, Shenzhen, China

**Keywords:** atopic dermatitis, *Malassezia globosa*, mouse model, T helper cells, thymic stromal lymphopoietin

## Abstract

Atopic dermatitis (AD) is a chronic and inflammatory disease with an immunogenetic basis that can be triggered by extrinsic and intrinsic factors, including dysbiosis of the skin microbiota. The lipophilic *Malassezia globosa* is one of the dominant fungal species on the skin of AD patients. *Malassezia* and the host pathophysiologic mechanism underlying its role in exacerbating AD symptoms remain to be elucidated. This experiment established a fungal overgrowth model by topical administration suspension of *M. globosa* on BALB/c mice (M group) and MC903-induced AD model (AD+M group). Our results suggested that more severe AD-like lesions and higher dermatitis scoring were observed in the AD+M group compared with the AD group. The expression of TSLP mRNA in the tissue and serum IgE were highly increased in the AD group, while decreased significantly in the AD+M group. The expression levels of IL-17A and IL-22 in ear tissues and serum were significantly increased with *M. globosa* stimulation, especially in the AD+M group. Meanwhile, the percentage of Th17 and Th22 cells in the spleen were positively correlated with IL-17A and IL-22 levels in the serum. In contrast, IFN-*γ* and IL-4 production were significantly decreased in the AD+M group compared with the AD group. This study demonstrated that overgrowing *M. globosa* could aggravate AD symptoms and that IL-17A and IL-22 may be involved in the process. The promotion of IL-17A and IL-22 production induced by *M. globosa* may restrain the development of TSLP and inhibit the Th1/Th2 type skin inflammation.

## 1. Introduction

Atopic dermatitis (AD) is a chronic, itchy, inflammatory skin disorder which affects up to 25% of children and 10% of adults [1, 2]. Multiple factors are implicated in the pathogenesis of AD, among which major factors involved are genetic susceptibility, dysbiosis of the skin microbiota, epidermal barrier abnormalities, and T cell–driven skin inflammation [3, 4].

The lipophilic yeast *Malassezia* is part of normal skin flora, but it is also recognized to play a role in inflammation of skin diseases [5]. *Malassezia globosa*, *Malassezia sympodialis*, *Malassezia slooffiae*, and *Malassezia restricta* are the most frequently found skin fungal commensals on human beings [6, 7]. Recent studies have shown that *M. globosa* and *M. sympodialis* are the dominant fungal species on the skin of Chinese, Japanese, and Korean AD patients [8–10]. The results of DNA sequencing analysis of skin mycobiome had shown that *M. globosa* and *M. restricta* accounted for around 77%–95% of the AD samples (*n* = 92) and 80%–95% of healthy controls (*n* = 48) [9]. The colonization rate is higher in AD lesion areas than in nonlesional areas, and the colonization rate is 2–5 times higher in severe AD patients than in mild-to-moderate AD patients, especially at sites of predilection such as head and neck [11, 12]. Furthermore, *Malassezia* can directly stimulate skin inflammation, and the skin barrier dysfunction of AD promote the release of *Malassezia* allergen [13, 14]. Glatz et al. [14] found that *Malassezia*-specific IgE is an important allergen-specific marker for severity of adult AD. *Malassezia* allergen, Mala s1, 5, 6, and 9 proteases, can activate mast cells to release inflammatory mediators and increase IgE-mediated degranulation via the TLR2/MyD88 signaling pathway. In addition, Mala s6, 11, and 13 have high homology with endogenous human proteins, causing crossreactivities with T cells.

Thymic stromal lymphopoietin (TSLP) is a critical proinflammatory cytokine in many allergic inflammatory diseases, which involved the release of T cell–attracting chemokines, promotion of type 2 helper T cell (Th2) responses and antimicrobial peptide activity on the skin [15]. *In vitro* and *in vivo* studies have provided strong evidence that TSLP is highly expressed in AD lesions. In mice, the overexpression of TSLP in the skin is sufficient to induce a disease phenotype characterized by all of the hallmark features of AD. Activated Th2 cells release IL-4 and IL-13, which could also synergize to induce TSLP expression by keratinocytes, suggesting a feed-forward inflammatory cascade. TSLP, IL-33, and the downstream Th2 cytokines are the common alarmins that are released in response to tissue damage, induce inflammation, and communicate with cutaneous sensory neurons to exacerbate pruritus. Corrigan et al. [16] found that TSLP was rapidly increased after cutaneous allergen challenge, leading to the recruitment and subsequent activation of dendritic cells (DCs). Notably, existing studies predominantly link *Malassezia*-induced TSLP to Th2 polarization, as evidenced by *M. globosa* activating lysophosphatidic acid receptors to induce keratinocyte-derived TSLP secretion [17]. However, emerging evidence suggests that fungal proteases may also modulate Th17-associated pathways. For instance, *Aspergillus* proteases were shown to induce ILC2 activation via the keratinocyte-derived TSLP/IL-33/IL-25 axis [18], yet whether *Malassezia* employs similar mechanisms to regulate Th17 responses in AD remains unclear.

Although previous studies have identified a role for *Malassezia* in AD, particularly through the promotion of IgE secretion and regulation of the Th2 response, the contribution of *M. globosa* in the regulation of the Th17-type response and its potential impact on TSLP expression has not been thoroughly explored. We hypothesize that *M. globosa* could aggravate the inflammation of AD via TSLP regulating downstream immune response of T helper cells. In the present study, we evaluated the effects *M. globosa* on the expression of TSLP and the differentiation of T helper cells using an inducible MC903 AD model. Our results demonstrated that *M. globosa* aggravated the lesions of AD and stimulated the proliferation of keratinocytes, and the expression of IL-17A and IL-22 in the tissue and in serum were enormously ascended under the stimulation of *M. globose*. However, the secretion of cytokines TSLP, IFN-*γ*, and IL-4 were inhibited. This dissociation between Th17 activation and TSLP inhibition challenges the prevailing paradigm of fungal-driven AD pathogenesis. It suggests a mechanism that may have been overlooked, which implies that *M. globosa* may stimulate the proliferation of keratinocytes through IL-17A-mediated signaling pathway in AD mice but not TSLP.

## 2. Materials and Methods

### 2.1. Mice

Then, 24 BALB/c mice (male, 7 weeks, 18-20 g) were purchased from SPF (Beijing) Biotechnology Co., Ltd. and fed in specific pathogen-free barrier conditions of a laboratory animal center of South China Agricultural University. Pre-experimental animals were arranged for 1 week of acclimization period. The experimental procedures were certified by the Animal Care and Use Committee of South China Agricultural University (Guangzhou, China).

### 2.2. Fungal Clinical Strain

*M. globosa* was isolated from a pityriasis versicolor patient at the Department of Dermatology, the Third Affiliated Hospital of Sun Yat-sen University, which was identified by morphology and ITS sequencing. For suspension preparation, the yeast was firstly grown at 31°C for 3 days on SDA medium containing rapeseed oil (1% peptone, 4% glucose, 0.01% yeast extract, 1% agar, 0.25% glycerol monostearate, 1% Tween 80, and 2% rapeseed oil) [19]. Single-cell suspensions were obtained by picking colonies after vortex washing in 0.01% Tween 20 in PBS, followed by 3000 rpm for 15 min, discarding the supernatant, and suspending *Malassezia* in olive oil and blow repeatedly. A standard curve of OD600 versus the number of viable yeast was established before the first modeling, and 10 gradients were constructed: 4.5–4.0–3.5–3.0–2.5–2.0–1.5–1.0–0.5–0.25, and hemocyte counting plate counts were performed to verify the actual CFU/mL; ultimately, a microtiter plate spectrophotometer (Biotek, EON, UT, United States) was used to adjust the Coccidioides cells suspended in olive oil solution to an optical density to 2 ODA600/100 *μ*l (≈2–10 × 10^6^ CFU) [20].

### 2.3. Experimental Model

Then, 24 BALB/c mice were randomly divided into 4 groups (*n* = 6 mice per group): the control group, AD group, *M. globosa* group (M group), and AD+M group. To induce AD-like model, 2 nmol MC903 (Sigma-Aldrich, MO, United States) solution was applied topically to bilateral ears skin for 7 consecutive days and then applied every other day to maintain modeling [21–23]. For overgrowth *M. globosa* with mice, 50 *μ*L yeast suspension prepared as above was applied topically onto the ears skin of the M group and AD+M group, respectively, from Day 8 to Day 15.

### 2.4. Assessment of Disease Activity in Mice

Changes in the ear thickness, dermatitis scoring, body weight, and the frequency of scratching were observed and recorded at four time points: Days 1, 8, 11, and 15. Ear thickness was monitored using a vernier caliper. The gross lesion of mice was evaluated by the dermatitis scoring index of AD based on their severity of edema, erythema, and hemorrhage (0, *none*; 1, *slight*; 2, *moderate*; and 3, *severe*) as described previously 22. Body weight was measured by animal weight balance. The number of scratches were recorded in each 10 min. On Day 15, around 0.3-cm^2^ ear tissue was cut and fixed in 4% paraformaldehyde and dehydrated and paraffin-embedded sections. Histopathological changes were observed by hematoxylin–eosin (H&E) staining, mast cell infiltration was counted by toluidine blue staining. The proliferation of keratinocytes was observed by anti-Ki67 fluorescence antibody staining.

### 2.5. Mycology Examination

Colonization of *M. globosa* was observed by Periodic acid Schiff (PAS) staining and fungal fluorescence staining under optical microscope and fluorescence microscope, respectively. Around 0.2 cm^2^ ear tissue were cut for CFU counting, ear tissue was ground into tissue suspension in the glass homogenizer and diluted to 10^−2^, 10^−3^, and 10^−4^. Then, 100 *μ*L *M. globosa* suspensions were coated in the solid medium, and each gradient was repeated three times. After labeling, they were incubated under 31°C for 3 days.

### 2.6. Quantitative Real-Time PCR

Transcription expression levels of IL-4, IFN-*γ*, IL-17A, and IL-22 were quantified by quantitative real-time PCR. Total RNA in ear tissues was extracted in accordance with product specification from Tissue RNA Kit (Omega Bio-tek, GA, United States). cDNA synthesis was adversely transcribed with total RNA and PrimeScriptTM RT master mix (Takara, Tokyo, Japan). RT-qPCR transcription reactions were performed with the primers (iGene Bio, Guangdong, China) and LC 480 SYBR Green I Master (Roche, Mannheim, Germany). The primer sequences used were as follows: *β*-actin: fwd 5⁣′-CCCTGAAGTACCCCATTGAAC-3⁣′, rev 5⁣′-CTTTTCACGGTTGGCCTTAG-3⁣′; IL-17A: fwd 5⁣′- GCTCCAGAAGGCCCTCAGA-3⁣′, rev 5⁣′-AGCTTTCCCTCCGCATTGA-3⁣′; TSLP: fwd 5⁣′-TCTGGAGATTGCATGAAGGA-3⁣′, rev 5⁣′-AGAGAAGCCCTCAATGACCA-3⁣′; IL-4: fwd 5⁣′-GAGGAGGAGGAGTTGGGTA-3⁣′, rev 5⁣′-AGATGCGGAAGGAGGAGGA-3⁣′; IFN-*γ*: fwd 5⁣′-AGGAACTGGCAAAAGGATGA-3⁣′, rev 5⁣′-GAGGGTAGGGTGAGGAGGA-3⁣′; and IL-22: fwd 5⁣′-ACAGGCCACAGGTTCTGGA-3⁣′, rev 5⁣′-TTGGCTCCAGGTTCATCCG-3⁣′.

### 2.7. Flow Cytometry

Fluorochrome-labeled antibodies were all obtained from Biolegend (Biolegend, CA, United States). Single-lymphocyte suspensions obtained from the fresh spleen were stimulated with cell activation cocktail. Extracellular molecular staining, fixation, permeabilization, and intracellular cytokine staining were carried out orderly. Fixed and intracellularly labeled cells were resuspend in cell staining buffer and detected on FACS Canto II (BD, CA, United States) immediately. The data were analyzed using Flowjo (10.8.1), and the gating strategy is described in Figure S1.

### 2.8. Quantification of Serum Cytokines

Quantification determination of mouse IL-4 (catalog M4000B-1), IFN-*γ* (catalog MIF00-1), IL-17A (catalog M1700-1), and IL-22 (catalog M2200) heterodimer concentration in plasma samples was analyzed by enzyme-linked immunosorbent assay using commercially available ELISA kits (R&D Systems, MN, United States), total serum IgE level detection was also used by ELISA kits (Cloud-Clone, Hubei, China), assay procedure according to manufacturer ´s instructions.

### 2.9. Statistical Analysis

Statistical significance was conducted using unpaired Student's *t*-test for direct comparisons of two independent groups, one-way or two-way ANOVA with Tukey's multiple comparison test as appropriate by GraphPad Prism 8.0 software. The value of *p* < 0.05 was considered statistically significant. Significance layout is showed as ⁣^∗^*p* < 0.05, ⁣^∗∗^*p* < 0.01, and ⁣^∗∗∗^*p* < 0.001.

## 3. Results

### 3.1. *M. globosa* Exacerbated Skin Inflammation in AD Mouse Model

**Figure 1:**
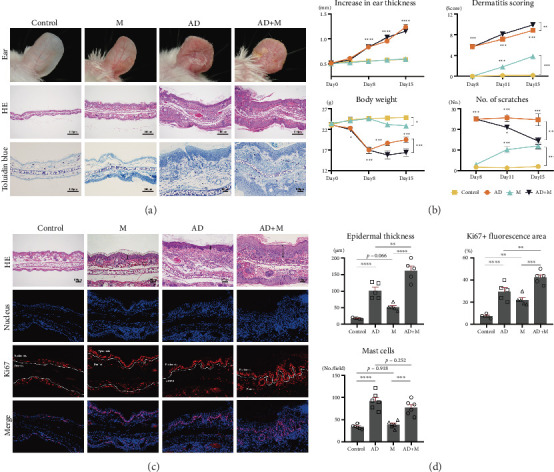
AD model was successfully induced and *Malassezia globosa* exacerbated its skin inflammation. (a) Macroscopic images of the ear and histology of ear sections stained with H&E and toluidine blue staining on Day 15. (b) Increase in ear thickness, dermatitis scoring, body weight change, and number of scratches were recorded on Days 0, 4, 8, 11, and 15. Lines showed the mean ± SEM of each group with 6 mice per group; statistics were calculated using repeated measures ANOVA. (c, d) Changes in epidermal thickness, mast cell infiltration, and Ki67+ fluorescence area in relation to total area of the epidermis on Day 15. In graphs C, three fields were randomly selected under the microscope from each slide, counting mast cells or measuring epidermal thickness. Statistical significance was determined with one-way ANOVA. Dotted line: basal membrane. ∗*p* < 0.05, ∗∗*p* < 0.01, and ∗∗∗*p* < 0.001.

The AD mouse model was successfully established by repeated topical application of MC903 according to a published protocol [21, 23–25]. In both the AD group and AD+M group, both ears of the mice showed significant erythema, edema, thicken, scaly, and telangiectasia (Figure 1a). In the M group, mild inflammation in both ears was also observed. The ear thickness of the AD group and AD+M group were significantly thicker than those of he control group and M group on Day 8 to Day 15 (*p* < 0.001). Dermatitis scoring in the M group and AD+M group were higher than those in the control group and AD group from Day 8 to Day 15 (*p* < 0.001). The body weight of the M group and AD+M group were lower than those in the control group and AD group (*p* < 0.05). Compared with the control group, the number of scratches in three experiment groups increased significantly (*p* < 0.001). However, in the AD+M group, scratching behavior weakened markedly on Days 11 and 15 (*p* < 0.05) (Figure 1b).

Histopathology of all three experiment groups showed hyperkeratosis, epidermal hyperplasia, and inflammatory cell infiltration in the dermis. In the AD+M group, obvious parakeratosis, acanthosis, hypergranulosis, and a mass of residual nuclei were also observed in the stratum corneum (Figure 1a). The infiltration of mast cells in the AD group and AD+M group was higher than that in the control group and M group (*p* < 0.001) (Figure 1a). The epidermis thickness in the AD+M groups was distinctly higher than that of the other three experimental groups (*p* < 0.01) (Figure 1c,d). Ki67+ fluorescence area of the epidermis in the M group and AD+M group were significantly larger than those of the control group and AD group (*p* < 0.01), mainly along the basal and granular layers (Figure 1c,d).

### 3.2. *M. globosa* Overgrow on the Mouse Skin

**Figure 2:**
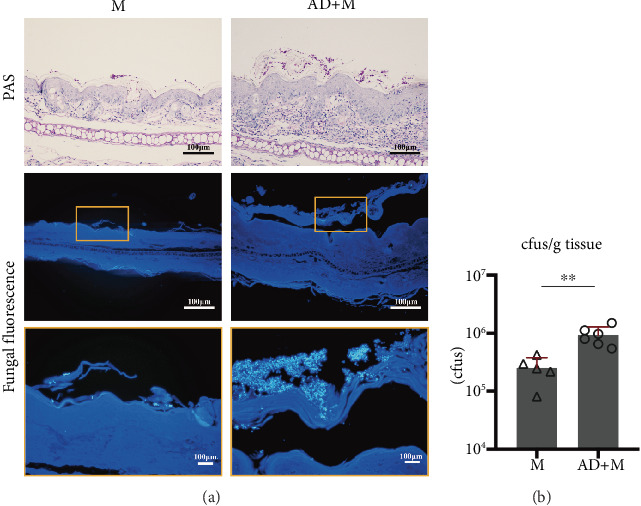
Overgrowth of *Malassezia globosa* in mouse ear tissues. (a) Ear sections stained with PAS or fungal fluorescence as indicated on Day 15. (b) Colony-forming unit of tissue per gram of ear. In graph B, a dilution with an average colony counts between 30 and 300 was selected to record. Every form represents one mouse, error bars denote mean ± SEM (n = 5–6). Statistical significance was determined with unpaired *t* test. ns, not significant; ∗*p* < 0.05, ∗∗*p* < 0.01, and ∗∗∗*p* < 0.001.

To study the interaction of *Malassezia* with AD *in vivo*, we established a mouse model of AD with overgrowth *M. globosa* by treating with suspension of *M. globosa* using olive oil. The results of PAS staining and fungal fluorescence staining showed that a mass of *M. globosa* yeast cells colonized in the stratum corneum of the M group and AD+M group, with the tendency of clustering (Figure 2a), and microscopically, the concentration of yeast cells in the AD+M group was significantly higher than that in the M group (Figure 2a). Consistently, CFU counting indicated that the colony-forming unit of *M. globosa* per gram of ear tissue in the AD+M group was statistically higher than that in the M group (*p* < 0.01) (Figure 2b).

### 3.3. Changes in Cytokine Expression Levels in Ear Tissue Exposure to *M. globosa*

**Figure 3:**
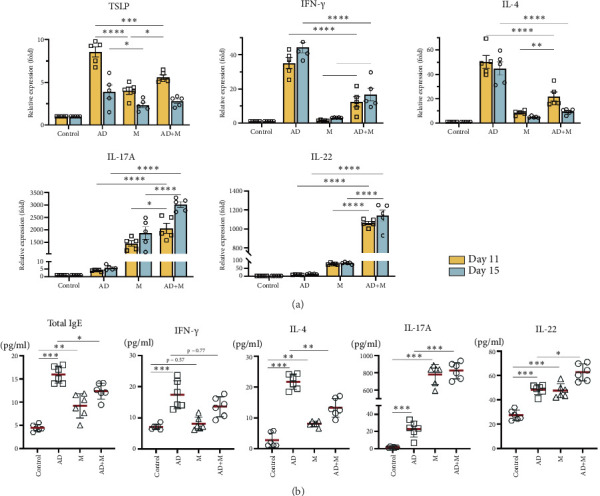
(a) Expression of IL-4, IFN-*γ*, and IL-17A mRNA in ear tissues. Transcription levels of cytokines TSLP, IL-4, IFN-*γ*, IL-22, and IL-17A in ear tissues of each group on Day 11 and Day 15. Graphs showed the mean ± SEM of each group with 6 mice per group. Statistics were calculated using one-way ANOVA. (b) Differential expression of cytokines and total IgE in serum. Total IgE, IL-4, IFN-*γ*, IL-22, and IL-17A plasma titres were measured by ELISA on Day 15. All graphs show pooled data from two to three independent experiments. Statistics were calculated using one-way ANOVA. ns, not significant; ∗*p* < 0.05, ∗∗*p* < 0.01, and ∗∗∗*p* < 0.001.

The expression of TSLP was robustly increased in ear tissues in three experimental groups on Day 11 and Day 15 compared with the control group, highest in the AD group, followed by the AD+M group and M group (*p* < 0.01). Interestingly, the level of TSLP on Day 15 in each experiment was significantly lower than that on Day 11 (*p* < 0.05) (Figure 3a).

A higher level of IL-4 and IFN-*γ* was observed on Day 11 in the AD group compared with the other three groups; though on Day 15, the trend of these two cytokines' AD group changed contrarily, with downregulating IL-4 (*p* < 0.01) and upregulation IFN-*γ* (*p* < 0.01). The expression fluctuant trend of IL-4 and IFN-*γ* in both the M group and AD+M group were similar with those in the AD group, but the levels were significantly lower than those in the AD group (*p* < 0.001) (Figure 3a).

In addition, our results showed that the IL-17A and IL-22 tissue level in the AD group and AD+M group on Day 11 and on Day 15 were significantly elevated than that of the control group and AD group (*p* < 0.001) (Figure 3a). All cytokine expression data are presented in the supporting information (available here).

### 3.4. Levels of Serum IgE and Th1/Th2/Th17 Cytokines Were Altered With Corresponding Changes in Splenocytes After Being Triggered by *M. globosa* in AD Mouse Model

The amount of total IgE ascended on Day 15 in three experimental groups, particularly in the AD group (*p* < 0.01). Serum IgE level was significantly higher in the M group compared with the control group, while it was distinctly lower in the AD+M group compared with the AD group (*p* < 0.01) (Figure 3b).

In the serum, we found the levels of IL-4 and IFN-*γ* were significantly higher in the AD group as compared to other three groups. IL-4 level was increased in the M group (*p* < 0.01) and there was no significant difference in IFN-*γ* (*p* = 0.937) when compared to the control group, but in the AD+M group, the colonization of *M. globosa* inhibited the secretion of IL-4. The serum IL-17A and IL-22 levels in three experimental groups on Day 15 were elevated than that from the control group (*p* < 0.05). Remarkably, Th17-polarized cytokines, IL-17 and IL-22, were excessively ascended in the M group and AD+M group, especially in the AD+M group (*p* < 0.001) (Figure 3b).

**Figure 4:**
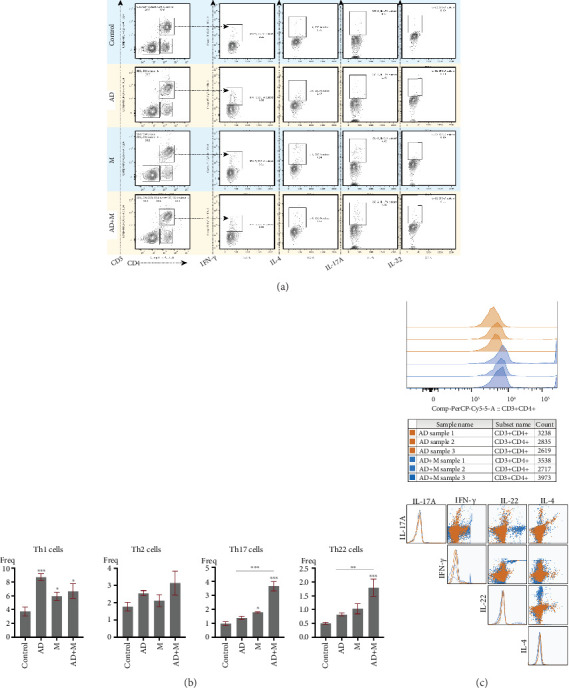
Representative FACS plots (a) and bar graph (b) show the FACS gating path and percentages of proliferating Th1 cells (CD3 + CD4+ IFN-*γ*+), Th2 cells (CD3 + CD4 + IL-4+), Th17 cells (D3 + CD4 + IL-17+), and Th22 cells (CD3 + CD4 + IL-22+). CD3+ and CD3 + CD4+ cells were sequentially circled from a single lymphocyte in the spleen. (c) Overlaid histograms show the expression levels of CD3 + CD4+ T cells in the AD group (orange) and AD+M group (blue). Each curve represents one biological replication. Scatterplot matrix showing the expression and co-expression of IL-17A, IFN-*γ*, IL-22, and IL-4 in CD3 + CD4+.

In the spleen tissue, the percentage of Th1 cells in CD3 + CD4 + T cells were increased during the three experimental groups on Day 15, especially in the AD group. There was no statistical difference in the proportion of Th2 cells between four groups. We also found the percentage of Th17 cells and Th22 cells was statistically ascended in the AD+M group compared to that in the AD group and M group (*p* < 0.01) (Figure 4a,b).

In the spleen tissue, the proportion of Th1 cells among CD3 + CD4+ T cells was increased on Day 15 in all three experimental groups, especially in the AD group. There was no statistical difference in the proportion of Th2 cells among the four groups. We also found that the proportions of Th17 cells and Th22 cells were statistically elevated in the AD+M group compared to the AD and M groups (*p* < 0.01) (Figure 4a,b). In addition, we compared the cytokine expression of the CD3 + CD4+ T cell population in the AD+M group with that in the AD group, focusing on the coexpression characteristics. The curve of the AD+M group was shifted to the right as a whole in histogram analysis, and the fluorescence intensity was higher in the CD3 + CD4+ cell population, indicating that more cells expressed CD3 + CD4+ cells, more IL-17A + IFN-*γ* + double-positive cells could be observed in the scatter plot, suggesting the expansion of the mixed Th1/Th17 phenotype, while the difference between the two groups in the Th2 (IL-4+) cell population was not significant (Figure 4c).

## 4. Discussion

The pathogenesis of AD is not fully understood, while skin barrier dysfunction is regarded as the initial step in the development of AD. Not only the itch–scratch cycle of AD can perpetuate skin barrier damage, dysbiosis of the skin and keratinocyte responses accelerate damage to the skin barrier [26]. Conversely, the impaired skin barrier of AD increases the risk of developing skin infections and promotes peicutaneous sensitization to external allergens. *Malassezia* species are by far the most common members of the microbiota on the human skin, while its role in AD remains not well understood. In the present study, we investigate the interplay of host–fungi using an inducible MC903 AD model colonized with overgrowth *M. globosa*.

Our results on experimental mice showed repeated MC903 topical administration for 7 days induced AD-like skin lesions on BALB/c mice, developed erythema and edema gradually, and appeared scaly and crusted finally. Consistent with other reports and the histological findings in AD, epidermal hyperplasia and hyperkeratosis were observed in MC903-treated skin with a massive cellular infiltration in the dermis. In the AD+M group, the overgrowth of *M. globosa* significantly aggravated the severity of these inflammatory features, showing obvious skin lesions as those observed in the AD group, and increasing dermatitis scoring. Considering the effect of clinical symptoms on the mental state of the mice caused weight loss and frequency of scratching reduced in the AD+M group, which suggests that *M. globosa* could stimulate a stronger skin inflammation in AD mice, similar to the clinical observation [11, 12]. Further mycological examination on ear tissues using fungal fluorescence stain and PAS stain confirmed that *M. globosa* were mainly observed in keratinized epidermis layer, especially clustered in the AD+M group with significantly higher numbers of positive stain yeast cells and CFU counts (*p* < 0.05). Unlike the experimental mouse model through disrupting the ear skin by tape stripping prior to infection [20, 24], we used olive oil as vehicle to make the suspension of *M. globosa* that keeps the high fungal load on the skin for more than 1 week without destroying the barrier integrity, which is a better model to study the interaction of host–*Malassezia*.

It appears that *Malassezia* interacts with the host directly via irritant pathways and indirectly through allergic response and immune pathways in AD. *Malassezia* itself may disrupt the integrity of the skin by releasing the unsaturated fatty acids which cause inflammation and irritation [27]. Sensitization to multiple *Malassezia* allergens may also enhance skin inflammation in AD [28]. Recent studies of the interaction of *Malassezia* with keratinocytes have highlighted their potential to modulate the immune response directed against them. In general, keratinocytes respond to the induction and propagation of inflammation by inducing cytokines produced by type 2 and type 17 immune cells. Moreover, keratinocytes produce cytokines such as TSLP, IL-25, and IL-33 following barrier disruption.

TSLP is regarded as hallmark of atopic diseases, which plays an important role in the development, maintenance, and progression of AD. It is a master regulator of Th2-driven inflammation as well as an activator of sensory neurons which directly evoke itch behaviors. Many studies showed that TSLP is highly expressed by keratinocytes in the AD lesions, but not in nonlesional skin or in serum samples [29–31]. Fungal products are one of the activators that induce the expression of TSLP from target cells. It is reported that *M. globosa* and *M. restricta* could induce the keratinocytes to secrete TSLP [17]. Consistent with the above notion, in this study, we found the expression levels of TSLP were significantly higher in three experimental groups compared with the control group. The AD group had the highest expression level of TSLP in Day 11 among the experimental groups, followed by the M group, then the AD+M group, which imply that *Malassezia* and epidermal barrier disruption having distinct ability to induce TSLP secretion from keratinocyte. Contrary to our expectation, the low expression level of TSLP was observed in the AD+M group compared to the AD group, which suggest that TSLP is not the key cytokines accentuate skin inflammation in the process of host-*Malassezia*, whereas the decrease in TSLP may be related to the high expression of Th17-like cytokines [20].

IL-17A/F are the signature cytokines of Th17 cell subset. It is well known that IL-17A plays an important role in the pathogenesis of diverse autoimmune and inflammatory diseases. Many studies demonstrate the presence of Th17 cells and IL-17 in AD patients [31, 32]. In recent years, Th17 signaling in AD has been better elucidated, with single-cell and transcriptome sequencing confirming the enrichment of Th17-related cytokines in AD lesional skin [33]. Elevated serum IL-17A/F levels in pediatric AD patients positively correlate with disease severity and early disease stage [34]. Secukinumab, an FDA- and EC-approved psoriasis biologic targeting IL-17A, is also in AD studies [35]. It has been reported that protective IL-17A responses could be observed in a variety of fungi, including *Candida*, dermatophytes, *Aspergillus*, and *Malazzesia*, etc. A very recent study reported by Sparber et al. [20] showed that *Malassezia* triggers a strong Th17 response with significant and rapidly induced expression of IL-17A and IL-22 transcripts that coordinates antifungal immunity and exacerbated skin inflammation. The inhibitory effect of Th17-type responses on TSLP is not the first time it has been observed. Sofia et al. showed that human IL-17A inhibited TSLP secretion by keratinocytes and enhanced the production of IL-8, but it neither directly affected the activation of DCs induced by TSLP-TSLPR nor influenced the promotion of Th2 cell differentiation by TSLP-DCs [36]. *In vitro* experiments demonstrated that dsRNA-induced TSLP expression in primary human keratinocytes was inhibited by IFN-*γ*, TGF-*β*, and IL-17 [37]. Xu et al. [38] found that IL-17A significantly inhibited TSLP production induced by double-stranded RNA (dsRNA) in human nasal epithelial cells, providing evidence for the opposing regulatory effects of IL-17A and IL-25 on TSLP in epithelial cells. In this study, we also found significantly higher expression levels of IL-17 and IL-22 in the tissue and in serum from three experimental groups as compared to the control group. Thus, we hypothesize that IL-17 is a key cytokine in AD that exacerbates skin inflammation during host–*Malassezia* action and may be overexpressed with some inhibitory effect on TLSP, IFN-*γ*, and IL-4.

In the M group and AD+M group, the expression of Ki-67 increased significantly in the ear tissues, mainly along the basal layers (Figure 2b). Ki-67 is a proven marker for cell proliferation, which is strongly present in psoriasis. Several studies reported *Malassezia* is associated with forming psoriatic skin lesions. As known, IL-22 is a critical cytokine in psoriasis via triggering pathological keratinocyte proliferation, and it also plays a role in the pathogenesis of AD by promoting epidermal barrier disruption and pruritus. However, the eruptions of the *Malassezia* groups showed AD-like lesions rather than psoriatic one. In AD lesions, IL-22 plays a more complex role, the expression of many genes encoding Th2 cell-associated (IL-4, IL-10, and IL-13) and Th22 cell-associated (IL-22) proteins are upregulated, which is mostly related to keratinocyte activity and T cell infiltration, a previous study has shown that IL-22 acts by activating keratinocytes and limiting their differentiation [39, 40]. Sparber et al. found that IL-17af^−^ deficiency attenuated mammalian skin thickening after exposure of barrier-impaired skin to *Malassezia*, but IL-22^−^ deficiency did not result in phenotypic changes. IL-17A enhances epidermal tight junction barrier integrity, and IL-17A influences the response to inflammation, epidermal differentiation, keratinocyte formation, and chemotaxis by affecting filaggrin mRNA expression, the production of functional filaggrin monomers, and their degradation at the level of enzymatic processing. By affecting *FLG* mRNA expression, the production of functional filaggrin monomers and their degradation at the enzymatic level of processing, IL-17A affects, in particular, responses to inflammation, epidermal differentiation, stratum corneum formation and stress, and chemotaxis, and the G/G genotype in IL-17A (rs2275913), together with coexisting mutations in the 2282del4 *FLG* gene, increases the incidence of AD by 9-fold [41]. The Th1 cell-associated and Th17 cell-associated cytokines seem to be overexpressed in chronic disease stages [42], particularly in Asian patients [43]. In this study, we found the overexpression of IFN-*γ* in the lesional tissue in the AD group. Though the main role of IFN-*γ* is to promote Th1 inflammatory response and inhibit Th2 inflammatory response, its role in the pathophysiology of AD is controversial. Recently, immunologic and clinical therapy research shows that the progression to chronic phase of AD is associated with an upregulation of Th22 and Th2 immune axes as well as significant increases in Th1-related products in the lesional skin. Interestingly, Zhang et al.'s single-cell sequencing results for AD found that Th2/Th22/Th17 correlated with localized disease severity at higher cell counts, but this was not the case for the expression of several of their marker genes, and it is clear that there are more complex regulatory mechanisms at work [40]. Reports of several clinical trials show promising results for patients with AD treated with oral or topical JAK inhibitor, although JAK transduces the signals of IFN-*γ* as well as Th2 cytokines. It seems that the presence of IFN-*γ* in the lesions may not necessarily be an inhibitor of Th2 immune response but may be involved in the pathogenesis of AD [44]. Furthermore, our results also showed that the expression levels of IFN-*γ* were significantly lower in the M group and AD+M group than in the AD group. Hence, the keratinocyte proliferation in the M group and AD+M group imply that IL-17 is also the key cytokine that aggravate *Malassezia*-treated skin inflammation.

In summary, our experiments showed that *M. globosa* might stimulate the proliferation of keratinocytes through IL-17A-mediated signaling pathways; inhibit the secretion of cytokines TSLP, IFN-*γ*, and IL-4; and selectively induce the polarization of the immune response toward Th17 cells. However, it remains to be elucidated exactly how IL-17 promotes a complex inflammatory condition in the lesional skin of AD. Despite the definitive role of keratinocytes in this loop, the complete pathway between the IL-23/IL-17 axis and the other components of the EIME (such as the microbiota and sensory nerves) has not yet been demonstrated. Our experimental model will facilitate the functional validation of such factors under diverse host conditions and thereby further promote a comprehensive understanding of the *Malassezia*–host interplay. This study also has some limitations. First, we used only male mice to minimize the influence of sex hormones on immune responses, but this may have underestimated immune differences in female mice. Future studies should consider the impact of sex differences. Second, although we observed a potential association between IL-17A/IL-22 and TSLP downregulation, the precise regulatory mechanism remains unclear and requires further validation through *in vitro* experiments or blocking assays.

## Data Availability

The data that support the findings of this study are available from the corresponding author upon reasonable request.

## Supporting Information

Supporting Information 1 Figure S1: Gating strategy for identification of IL-17A and IL-22 producing CD4 + T cells by flow cytometry. CD3 + CD4 + T cells were gated from the lymphocyte population, within the CD3 + CD4 + T cell subset, expression of IL-17A and IL-22 was analyzed by intracellular cytokine staining. Representative dot plots show the frequencies of IL-17A+ and IL-22+ cells within the CD4+ T cell population. Histograms on the right display the proportion of IL-17A+ and IL-22+ cells, respectively.

Supporting Information 2 Supporting file: Data on ear tissue, serological cytokine expression, and splenic Th cell percentage for each mouse sample.
