# Supplementary figures and images for: Effects of Malassezia globosa on the Expression of Thymic Stromal Lymphopoietin and Differentiation of T Helper Cells in MC903-Induced Atopic Dermatitis Mouse Model
Source: Int J Microbiol. 2025 Apr 22;2025:3586621. doi: 10.1155/ijm/3586621 (PMC12041643; doi:10.1155/ijm/3586621)

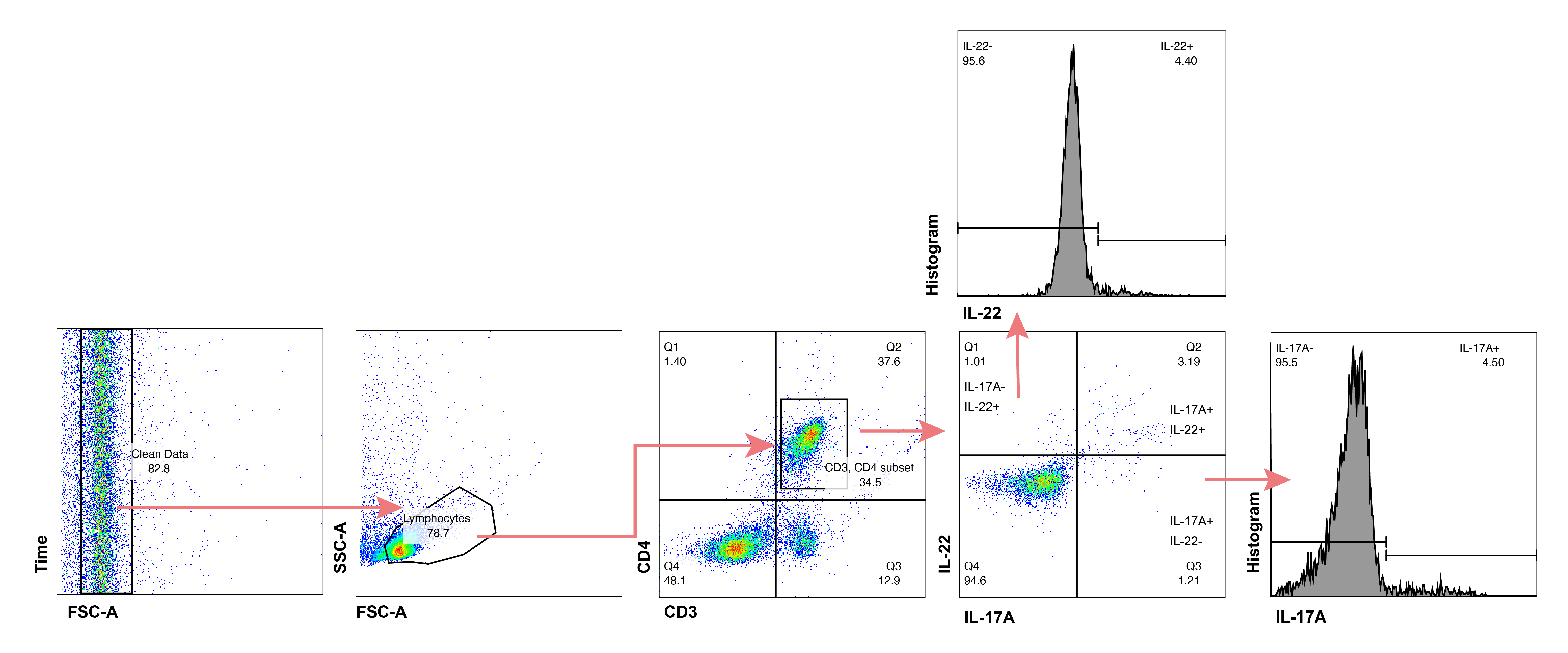

Supplement: Supporting Information 1 — Figure S1: Gating strategy for identification of IL-17A and IL-22 producing CD4 + T cells by flow cytometry. CD3 + CD4 + T cells were gated from the lymphocyte population, within the CD3 + CD4 + T cell subset, expression of IL-17A and IL-22 was analyzed by intracellular cytokine staining. Representative dot plots show the frequencies of IL-17A+ and IL-22+ cells within the CD4+ T cell population. Histograms on the right display the proportion of IL-17A+ and IL-22+ cells, respectively. [file 3586621.f1.png]
